# Contrasting effects of tree species and genetic diversity on the leaf-miner communities associated with silver birch

**DOI:** 10.1007/s00442-019-04351-x

**Published:** 2019-02-24

**Authors:** Sandra Barantal, Bastien Castagneyrol, Walter Durka, Glenn Iason, Simon Morath, Julia Koricheva

**Affiliations:** 1 School of Biological Sciences, Royal Holloway University of London, Egham, Surrey TW20 0EX UK; 2 Ecotron-CNRS, 1 Chemin du Rioux, 34980 Monferrier, France; 3 BIOGECO, INRA, Univ. Bordeaux, 33610 Cestas, France; 4 Helmholtz Centre for Environmental Research-UFZ, 06120 Halle, Germany; 5 James Hutton Institute, Aberdeen, AB15 8QH UK; 6 Forest Research, Alice Holt Lodge, Farnham, Surrey GU10 4LH UK

**Keywords:** Species diversity, Genetic diversity, Leaf miners, Boreal forest

## Abstract

Both species and genetic diversity of plant communities can affect insect herbivores, but a few studies have compared the effects of both diversity levels within the same experimental context. We compared the effects of tree species and genetic diversity on abundance, species richness, and β-diversity of leaf-miner communities associated with silver birch using two long-term forest diversity experiments in Finland where birch trees were planted in monocultures and mixtures of birch genotypes or other trees species. Although both abundance and species richness of leaf miners differed among birch genotypes at the tree level, birch genetic diversity had no significant effect on miner abundance and species richness at the plot level. Instead, birch genetic diversity affected leaf-miner β-diversity with species turnover being higher among trees within genotypic mixtures than among trees within monoclonal plots. In contrast, tree species diversity had a significant negative effect on both leaf-miner abundance and species richness at plot level, but no effect on miner β-diversity. Significant tree species diversity effects on leaf-miner abundance and species richness were found only in plots with high tree density. We have demonstrated that plant species and genetic diversity play important but contrasting roles in structuring associated herbivore communities. Tree species diversity largely affects miner abundance and species richness, whereas tree genetic diversity affects miner β-diversity. These results have important implications for conservation and management of woodlands.

**Electronic supplementary material:**

The online version of this article (10.1007/s00442-019-04351-x) contains supplementary material, which is available to authorized users.

## Introduction

Plant species diversity has been long recognised as an important determinant of the abundance and species richness of organisms at higher trophic levels (Elton 1958; Hutchinson 1959; Root 1973; Hunter and Price 1992). More recently, plant genetic diversity has also been shown to have significant effects on consumer communities (Bailey et al. 2009; Tack and Roslin 2011; Castagneyrol et al. 2012; Barton et al. 2015; Koricheva and Hayes 2018). Therefore, the ongoing rapid losses of both plant species and genetic diversity in natural and managed ecosystems are likely to affect the associated communities of herbivores. Although losses of plant species and genetic diversity in natural and managed habitats usually occur in parallel, only a few studies to date have simultaneously compared the effects of plant genotypic and species diversity on arthropods. While some of these studies have reported stronger effects of plant species diversity on herbivores (Campos-Navarrete et al. 2015; Abdala-Roberts et al. 2015), others found plant genetic diversity effects to be equally strong (Cook-Patton et al. 2011; Moreira et al. 2014), stronger (Crawford and Rudgers 2013) or opposite (Hahn et al. 2017) to species diversity effects. A recent meta-analysis comparing the magnitudes of plant genetic and species diversity effects on arthropods found similar effects of these two facet of plant diversity, but only 16 out of 60 studies included in the analysis allowed a direct comparison between genetic and species diversity effects (Koricheva and Hayes 2018). Therefore, more studies which simultaneously quantify the effects of plant species and genetic diversity within the same experimental context are needed to improve our abilities to predict the impacts of plant diversity loss on herbivore communities.

Plant diversity effects are likely to differ depending on the herbivore response variable studied (Kambach et al. 2016) as well as between specialist and generalist herbivores (Castagneyrol et al. 2014). Abundance of specialist herbivores is usually negatively related to plant species diversity due to decrease in density of host plants (dilution effect) and increase in the frequency of non-host plants in diverse stands, which might cause physical or olfactorial masking (Jactel and Brockerhoff 2007; Kostenko et al. 2012; Abdala-Roberts et al. 2015). Similar decreases in abundance of specialist herbivores may be observed with increase in plant genetic diversity if herbivores in question display preferences for particular plant genotypes (Peacock and Herrick 2000; Utsumi et al. 2011; Tooker and Frank 2012). The host plant density often covaries with the frequency of non-host plants in experiments manipulating plant diversity, but recent work has demonstrated the importance of manipulating plant density and frequency independently to provide deeper insight of how plant neighborhood influences host colonisation by specialists (Kim and Underwood 2015). In contrast to specialist herbivores, generalist herbivores are less sensitive to the alteration in host plant density and may benefit from a greater plant diversity in mixed stands via diet mixing and spillover effects (White and Whitham 2000; Unsicker et al. 2008; McArt and Thaler 2013).

In terms of herbivore species richness, diverse plant communities are likely to harbour more species of specialist herbivores by providing more niches (Hutchinson 1959; Lewinsohn et al. 2005). Most of studies linking plant and herbivore species diversity are conducted at a plant community level, whereas the species richness of herbivores associated with a focal plant species along a plant species diversity gradient have been less explored (but see Campos-Navarrete et al. 2015). Herbivore species richness has also been shown to be higher in more genetically diverse plant stands (Koricheva and Hayes 2018). Finally, plant diversity may also alter the species composition of herbivore communities, but such compositional changes are less often explored than changes in herbivore abundance and species richness. Assuming some degree of specialization of herbivores on particular species or genotypes, herbivores are likely to show higher compositional dissimilarity between monoculture stands containing plant species or genotypes preferred only by particular herbivore species than between mixed stands containing plant species or genotypes preferred by several species of herbivores (Bangert et al. 2006; Whitham et al. 2012).

Here, we compare the effects of tree species and genetic diversity on leaf-miner communities on silver birch (*Betula pendula* Roth) using two long-term experiments in Satakunta, Finland. Silver birch is the most abundant and economically important broadleaf tree species of the Finnish boreal forests which hosts a large number of species of insect herbivores (Shaw 1984; Atkinson 1992) and is known to display considerable genetic variation in leaf traits (Laitinen et al. 2000). We have separately manipulated silver birch density and species diversity, as well as birch genetic diversity at the same spatio-temporal scale, thereby allowing a comparison of genetic and species diversity effects. Leaf miners provide an excellent study system for exploring these effects, notably because this trophic group contains a diverse range of species most of which are specialists and, hence, should be able to distinguish between host plant species and genotypes. The main objective of our study was to compare the effects of tree species and genetic diversity on birch leaf-miner abundance, species richness (α-diversity), and the β-diversity of leaf-miner communities (i.e., the variation in species composition among sampling units). In the species diversity experiment, we further explored the potential interaction between species diversity and tree density.

## Materials and methods

### Experiments

We used the tree species diversity experiment and the birch genetic diversity experiment established in Satakunta, SW Finland (61°N 21°E) in 1999–2000 (http://www.sataforestdiversity.org/).

The tree species diversity experiment (SDE) was established on three clear-cut areas located 10–30 km from each other. Each area contains 38 plots (20 × 20 m) planted with monocultures and different two, three, and five species mixtures of silver birch (*Betula pendula*), Scots pine (*Pinus sylvestris*), Norway spruce (*Picea abies*), Siberian larch (*Larix sibirica*), and black alder (*Alnus glutinosa*) (Online Resource 1). Species mixtures were composed to create a gradient from purely coniferous stands through mixed deciduous and coniferous stand to purely broadleaf ones. Only treatments including birch were selected in this study to facilitate the comparison with birch genetic diversity experiment, resulting in nine different species composition treatments which included birch monocultures and birch/alder, birch/pine, birch/spruce, birch/pine/spruce, birch/pine/larch, birch/pine/alder, birch/larch/alder, and birch/pine/spruce/larch/alder mixtures (Online Resource 1). Each area includes two replicates of each species composition treatment and trees within plots were planted at 1.5 m intervals (169 trees per plot). The different tree species are planted in equal proportions in mixtures and the position of individual trees within mixed plots is randomized. Tree seedlings originated from a local tree nursery and are genetically diverse.

The birch genetic diversity experiment (GDE) was established on a clear-cut area located 14 km and 39 km from the closest and the furthest SDE areas, respectively. It includes forty-eight 20 × 20 m plots planted with eight different genotypes of silver birch (V5818, V5952, JR¼, 36, K2674, K1659, O154, and K5834). These silver birch genotypes have southern Finnish origin (61–63°N) and have been obtained by micropropagation of vegetative buds of mature trees. They are known to differ in their growth and leaf characteristics as well as in resistance to herbivores and pathogens (Viherä-Aarnio and Velling 2001; Poteri and Saikkonen 2001; Barton et al. 2015). The genotypes were planted in monoclonal plots and in different mixtures as follows: two-genotype mixtures (five different combinations), four-genotype mixtures (five different combinations), and eight-genotype mixtures (Online Resource 1). Trees within plots were planted at 2 m intervals (100 trees per plot) with position of each genotype randomized. Planting intervals were different in GDE compare to SDE, because birch grows faster than the other target species in SDE; hence, a larger inter-tree distance in GDE was chosen to allow for roughly the same intensity of interactions between trees as in SDE. Monocultures of five out of eight genotypes are replicated two–three times, but, for the remaining three genotypes, only a single monoculture plot was planted due to the shortage of micropropagated material. Each particular genotype mixture is replicated two-to-six times within the area.

The SDE and GDE were thinned in 2013 to reduce tree density by half while keeping proportions of the different species/genotypes equal. In the SDE, one replicate of each treatment per area was left unthinned to offer the opportunity to compare tree species diversity effects at two different tree stand densities, hereafter low tree density (on average 83 trees per plot) and high tree density (on average 116 trees per plot) treatments.

### Clone genotyping

Birch leaves were sampled from three trees of each genotype in monocultures in GDE in 2014. Leaf samples were genotyped at nine microsatellite loci developed for silver birch (Kulju et al. 2004, Online Resource 2). Samples were assigned to a particular reference genotype when at least five out of nine loci were identical, thus allowing for some genotyping error and ambiguous genotypes. These preliminary analyses had revealed that while seven out of eight genotypes had a very low error rate, samples of genotype K1659 showed different multilocus genotypes. Thus, we sampled and genotyped all K1659 trees in 2015, revealing that most of them in fact belonged to genotype V5952 (80%), only 17% to K1659 and 3% to other genotypes, likely due to labelling errors during the micropropagation. For the analyses presented here, we used the verified genotypes; the genotype composition was modified accordingly (Online Resource 1).

### Leaf-miner sampling

Leaf mines were surveyed in 2011 and 2014, in both the early and late season (June and August), because the leaf-miner community composition on birch changes over the growing season (Vehviläinen et al. 2006). Mines from June were unlikely to be recorded in August as leaf mining is known to cause premature leaf abscission (Zvereva and Kozlov 2014), and hence, most of the leaves with the early season mines would have abscised by the time of the late season monitoring.

Ten trees per plot were sampled in the SDE in 2011 and five trees per plot in 2014. In the GDE, five trees per genotype per plot were sampled in both 2011 and 2014. Birch tree height was measured only in SDE in 2011 and was on average 8.40 m. For each individual tree, four branches with 50 leaves each from low to mid canopy were randomly sampled and leaves were examined for the presence of mines. The different miner species were identified using the ‘Plant Parasites of Europe’ (http://www.bladmineerders.nl) and the ‘British Leaf Miners’ websites (http://www.leafmines.co.uk/index.htm). Using the above sources, each miner species was classified as either a specialist (feeds on genus *Betula* only) or a generalist (able to feed on the other genera of broadleaf tree species, including black alder which was planted in the SDE) (Online Resource 3). The numbers of mines per species were summed across the four branches for each tree.

### Data analysis

#### Leaf-miner abundance and species richness (α-diversity)

We used mixed-effects models to analyse the effects of tree species and genetic diversity on leaf-miner abundance and species richness (α-diversity) at both tree level and plot level. Because of the unbalanced proportion of the two birch genotypes in GDE, K1659, and V5952, we used the Shannon index of genetic diversity (hereafter genetic diversity) instead of genotype richness as a measure of plot genetic diversity. To make it comparable with SDE, we also used the Shannon index of species diversity (hereafter species diversity) instead of species richness. The Shannon index was strongly correlated with genotype and species richness (*r* = 0.94 and *r* = 0.96, respectively).

To conduct plot-level analysis, sample-based rarefaction was used to make the sampling effort (number of trees sampled) comparable for all plots and years in SDE and GDE. Rarefaction was performed with the *rich* package in *R* (Rossi 2011), setting a sample size of *N* = 5, which corresponds to the minimal number of trees sampled per plot in both experiments. Linear models were used at plot level, because rarefaction resulted in a continuous distribution of data. Different models were used for the SDE and GDE because of the additional tree density treatment in the SDE. Miner abundance and species richness data were square root transformed to improve the homogeneity of variance and normality when needed. For the SDE, we ran models separately for each sampling year as the tree density effect (high density in unthinned plots vs low density in thinned plots) was included only in 2014 following thinning in 2013. For each year, linear mixed-effects models included tree species diversity as fixed factor in interaction with season; the tree density was included as fixed factor in interaction with others fixed factors in the 2014 model. Area (the three different experimental sites) was included as an additional fixed factor and plot was included as a random factor. We also tested for the effects of tree species diversity on the abundance of generalist and specialist leaf miners separately. For the GDE, we performed linear mixed-effects models with genetic diversity in interaction with year and season as fixed factors and with plot as a random factor to take into account repeated measurements across seasons.

We additionally ran a generalized linear mixed-effects model (GLMM) at tree level to analyse the effect of genotype identity in interaction with genetic diversity, season, and year as fixed factors. A negative binomial distribution was chosen because of the overdispersion of count data (Zuur et al. 2009). To take into account the temporal autocorrelation (repeated measures across seasons) and the spatial autocorrelation (individual trees within plot), we used tree ID nested within plot as a random factor. As the K1659 genotype was not well represented across the diversity gradient, it was excluded from the model.

For all the models, the non-significant interaction terms were sequentially removed from the models and the analysis were run again until all remaining terms of interaction were significant.

#### Leaf-miner β-diversity

We calculated the overall β-diversity (βsor) within plots and across plots, and partitioned it into components of species turnover (βsim) and species nestedness (βnes), following the approach of Baselga (2010). A typical spatial species turnover pattern occurs when the species at one site are replaced by different species at another site. Nestedness occurs when the species pool present in one site is a subset of that of another more species-rich site. We also computed abundance-based dissimilarity indices that account for variation in abundance (Baselga 2016). Abundance-based assemblage dissimilarity (βbray) can be partitioned into two components, either balanced variation in abundance (βbal) or abundance gradients (βgra). Balanced variation in abundance occurs when the individuals of some species in one site are substituted by the same number of individuals of different species in another site. An abundance gradient is a pattern whereby some individuals are lost from one site to the other.

All these indices were first computed at plot level within each experiment, separately within monocultures and within polycultures (and separately for each year and each season). Because, in each experiment, the number of mixtures was higher than the number of monocultures, these indices in polycultures were computed by resampling the same number as numbers of monocultures present in each experiment. The differences among mono- and polycultures were assessed by a paired *t* test (using the β-diversity indexes within year and season as temporal replicates). We also computed the within-plot beta-diversity indices for each year and each season using data at tree level. These indices were computed by resampling the same number of trees for each plot (i.e., *N* = 5). We performed linear mixed-effects models with tree diversity (species or genetic diversity) in interaction with year and season as fixed factors and with plot as a random factor. For the SDE, the area was included as an additional fixed factor. As the thinning effect was not significant in the SDE, this factor was deleted and both years were analysed within the same model.

## Results

**Fig. 1:**
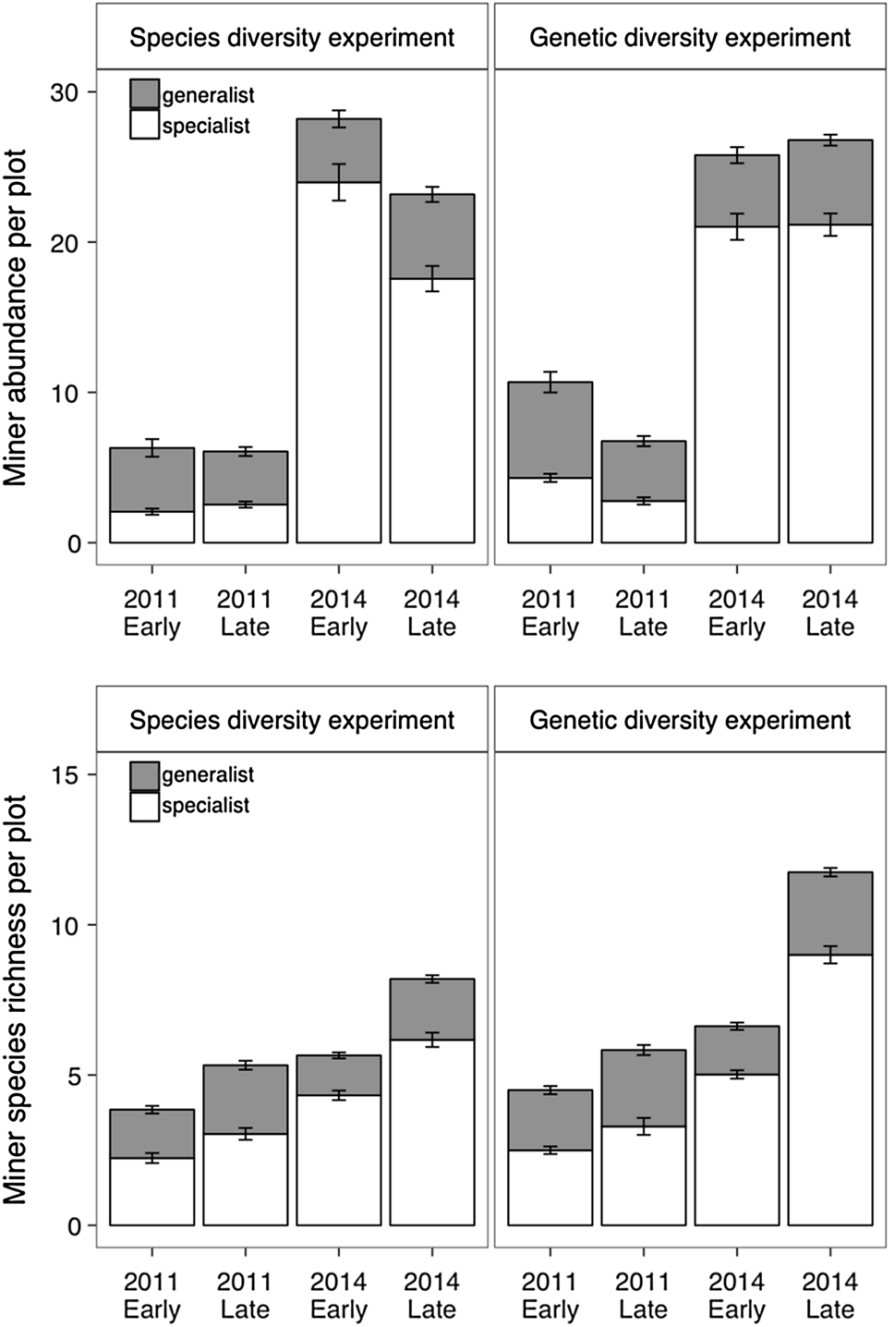
Birch leaf-miner abundance (top panel) and species richness per plot (bottom panel), calculated as the sum of mines across five sampled trees (mean per plot ± SE) within each season (early season: June; late season: August) and year in the species diversity experiment (SDE) and in the genetic diversity experiment (GDE)

In total, 11,506 leaf mines which belonged to 28 different species were recorded on birch trees over the course of the study (Online Resource 1). The leaf-miner abundance and species richness significantly differed between years and seasons in both experiments (Tables 1, 2, Fig. 1). Overall, the total leaf-miner abundance and species richness were higher in 2014 than in 2011 for both experiments (Fig. 1). Among the 28 recorded miner species, eight were generalists, whereas the other species were *Betula* specialists (Online Resource 1). The abundance of specialist leaf miners was higher in 2014 than in 2011 for both experiments, whereas generalist abundance did not differ between years (Fig. 1).

**Table 1:** Results of linear mixed-effect models testing for the effects of tree species diversity and season within each year on birch leaf-miner abundance and species richness at plot level (using rarefied data with five randomly sampled trees per plot)

	Miner abundance			Miner richness		
	*df*	*χ* ^2^	*P*	*df*	*χ* ^2^	*P*
*2011*						
Species diversity (SD)	1	0.48	0.49	1	0.63	0.43
Season (S)	1	0.009	0.92	1	10.41	**0.0013**
Area	2	4.08	0.13	2	3.16	0.21
*2014*						
Species diversity (SD)	1	4.35	**0.037**	1	1.79	0.18
Season (S)	1	8.83	**0.003**	1	55.74	**< 0.001**
Stand density (D)	1	0.01	0.92	1	0.72	0.40
Area	2	0.29	0.86	2	0.99	0.61
SD:D	1	5.79	**0.016**	1	5.24	**0.02**

In 2014, the stand density treatment was included. Only significant interactions were kept in the final model. Significant effects (*P* < 0.05) are in bold

**Table 2:** Results of linear mixed-effect models testing for the effects of genetic diversity, season, and year on birch leaf-miner abundance and species richness at tree level (including genotype identity effect) and at plot level (using rarefied data with five randomly sampled trees per plot)

	Tree level				Plot level		
	*df*	*χ* ^2^	*P*		*df*	*χ* ^2^	*P*
*Miner abundance*							
Genetic diversity (GD)	1	11.12	**< 0.001**	Genetic diversity (GD)	1	0.52	0.47
Genotype identity (G)	6	50.73	**< 0.001**	Season (S)	1	20.95	**< 0.001**
Season (S)	1	9.06	**0.003**	Year (Y)	1	503.90	**< 0.001**
Year (Y)	1	1106.17	**< 0.001**	S:Y	1	19.71	**< 0.001**
GD:S	1	14.73	**< 0.001**				
G:S	1	26.03	**< 0.001**				
S:Y	1	42.65	**< 0.001**				
*Miner species richness*							
Genetic diversity (GD)	1	1.98	0.16	Genetic diversity (GD)	1	1.73	0.19
Genotype identity (G)	6	50.85	**< 0.001**	Season (S)	1	66.68	**< 0.001**
Season (S)	1	266.51	**< 0.001**	Year (Y)	1	254.75	**< 0.001**
Year (Y)	1	796.7	**< 0.001**	S:Y	1	43.09	**< 0.001**
G:Y	1	13.24	**0.039**				
S:Y	1	21.58	**< 0.001**				

Only significant interactions were kept in the final model. Significant effects (*P* < 0.05) are in bold

### Tree diversity effects on leaf-miner abundance and species richness (α-diversity)

**Fig. 2:**
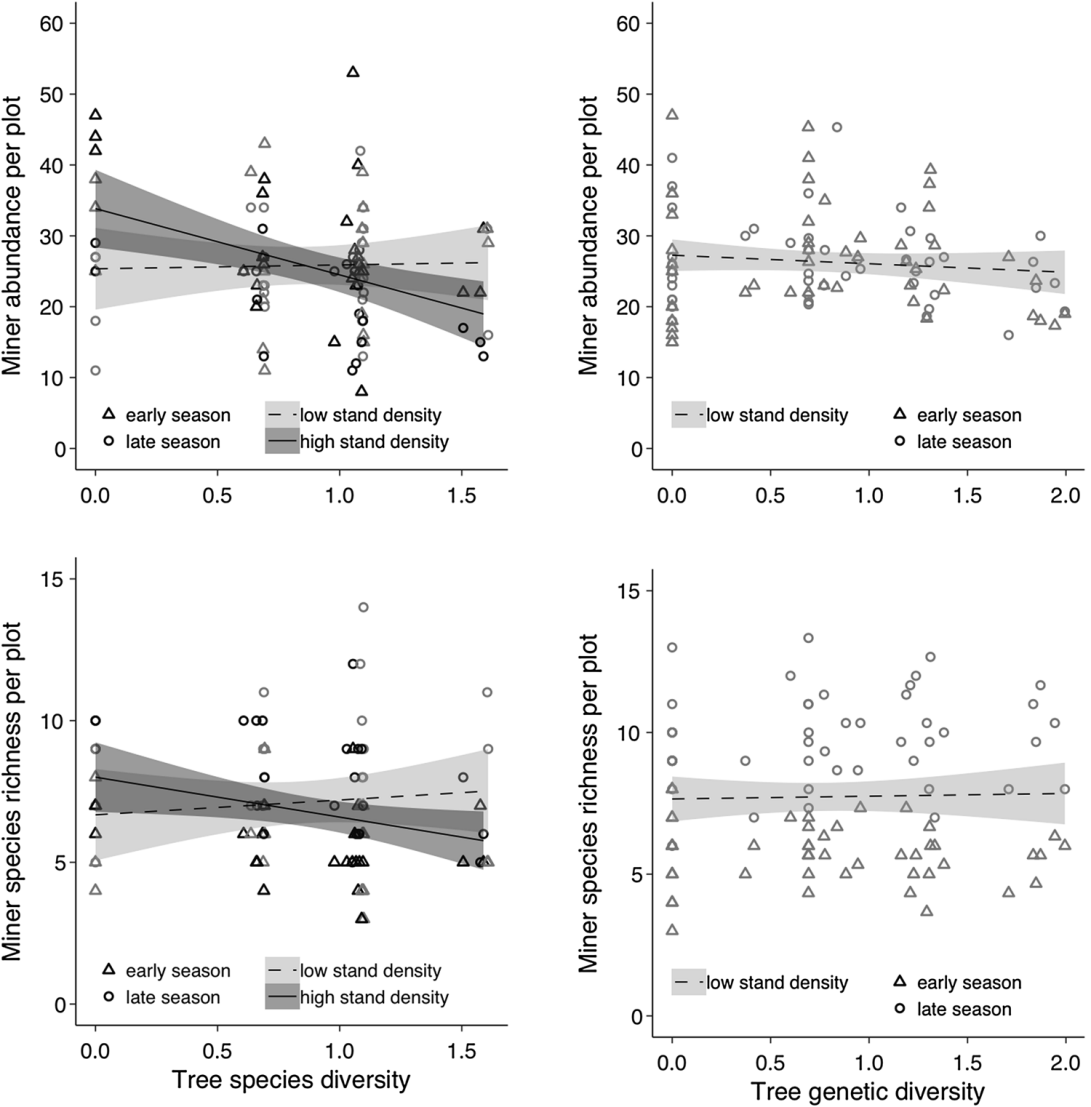
Birch leaf-miner abundance and species richness per plot (calculated as the sum across five sampled trees) as function of tree species diversity (right panels) and genetic diversity (left panels) in the SDE and in GDE (only 2014 data are displayed). Tree diversity is calculated using the Shannon index. Triangles symbols indicate data in the early season and circles indicate data in late season. Grey symbols indicate data in low stand density plots and black symbols indicate data in high stand density plots. Solid lines represent significant relationships (*P* < 0.05); dashed lines represent non-significant relationships (*P* > 0.05)

In 2011, leaf-miner abundance on birch was not affected by tree species diversity (Table 1). In 2014, leaf-miner abundance significantly decreased with increasing tree species diversity but only in high-density plots (i.e., in unthinned plots) (Table 1, Fig. 2). A decrease in miner abundance with tree species diversity was observed only for the specialists (model coefficient = − 4.88 ± 3.37, *P* = 0.003) but not the generalists (model coefficient = 2.5 ± 1.81 *P* = 0.96). Similarly to miner abundance, species richness of leaf miners was not affected by tree species diversity in 2011, but decreased with tree species diversity in high-density plots in 2014 (Table 1, Fig. 2).

**Fig. 3:**
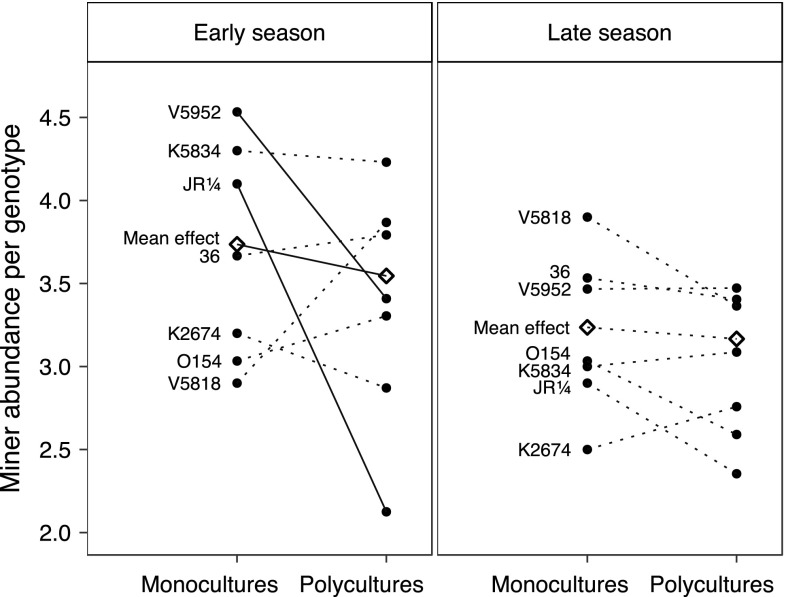
Birch leaf-miner abundance per genotype (black circles) and mean abundance across genotypes (white diamonds) in monocultures and polycultures of the genetic diversity experiment in early and late season; data are averaged across years. Solid lines represent significant differences between mono- and polycultures (*P* < 0.05)

**Fig. 4:**
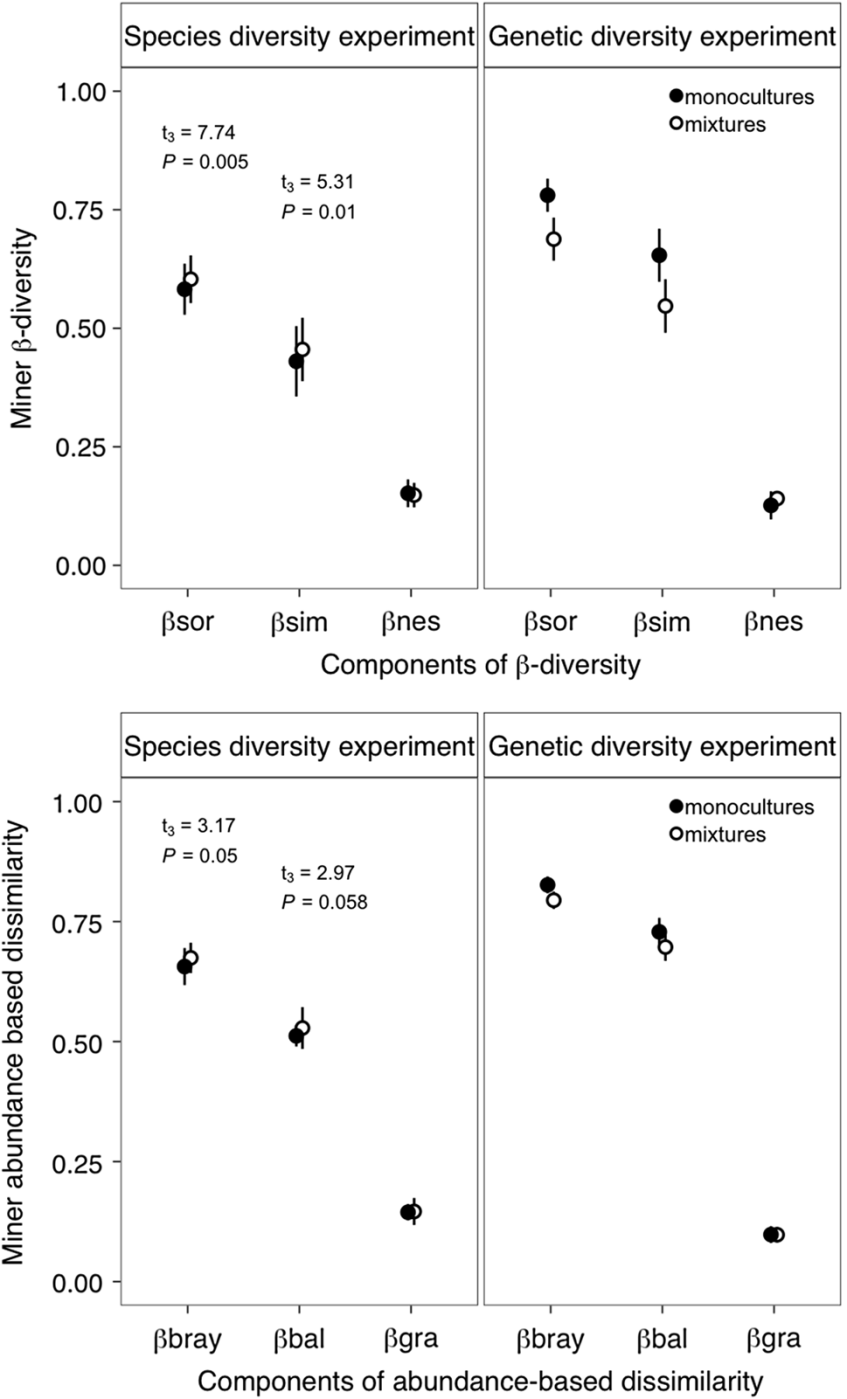
Leaf-miner β-diversity (means ± SD) in the genetic diversity and species diversity experiments based on the presence/absence of miner species (top panel) and abundance of miner species (bottom panel). βsor: overall β-diversity (βsor), βsim: species turnover, βnes: species nestedness, βbray: overall abundance-based assemblage dissimilarity, βbal: balanced variation in abundance, βgra: abundance gradients

The birch genetic diversity did not affect leaf-miner abundance or species richness when analyses were done at the plot level (Table 2, Fig. 2). Analyses at the tree level revealed that leaf-miner abundance significantly differed between birch genotypes (Table 2), but the genotype effect depended on season (Table 2, Fig. 4). The leaf-miner abundance at the tree level was lower on plots with higher genetic diversity, but only in the early season (Table 2, Fig. 3). There was no significant interaction between genetic diversity and genotype identity, but only two genotypes in the early season exhibited a significant decrease in miner abundance with increase in genetic diversity (V5952 and JR1/4, Fig. 3). Leaf-miner species richness was affected by genotype identity in interaction with year but not by genetic diversity (Table 2).

### Tree diversity effects on leaf-miner β-diversity

In both GDE and SDE, differences in miner species composition among the plots were largely explained by the turnover component βsim rather than by the nestedness component βnes (Fig. 4). The abundance variation (βbray) across plots was mainly driven by balanced variation (βbal) rather than by an abundance gradient (Fig. 4).

**Fig. 5:**
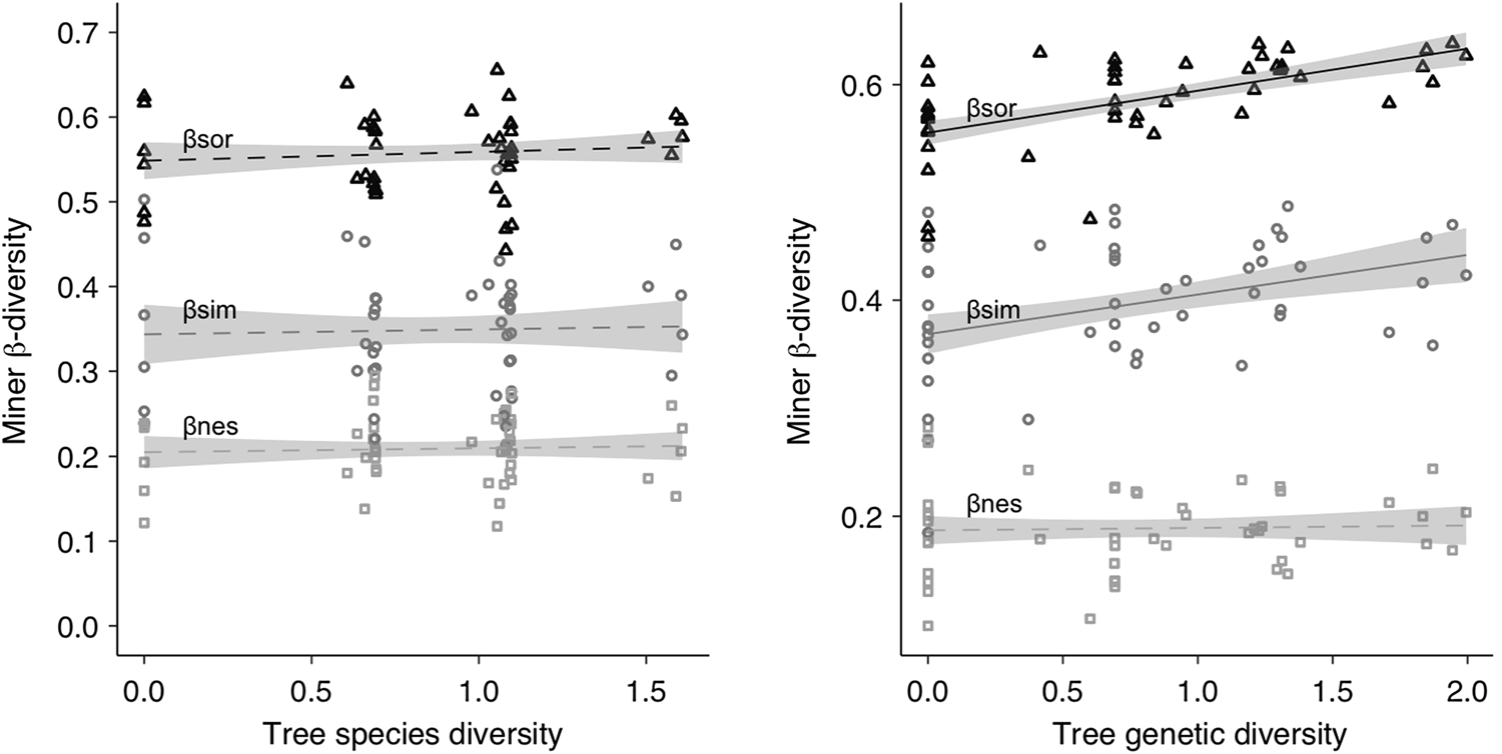
Leaf-miner β-diversity at tree level in SDE and GDE as function of tree species diversity (left panel) and tree genetic diversity (right panel). Data were averaged across years and season (and tree density treatment in the species diversity experiment). βsor: overall β-diversity (βsor), βsim: species turnover, βnes: species nestedness. Solid lines represent significant relationships (*P* < 0.05), dashed lines represent non-significant relationships (*P* > 0.05)

In SDE, the components of β-diversity did not differ between monocultures and polycultures at the stand level (Fig. 4). Similarly, the within-plot β-diversity did not change across the species diversity gradient (Table 3, Fig. 5). In GDE, the overall β-diversity (βsor) and the βsim component at the stand level were significantly higher among monoclonal plots compared to polyclonal plots for both years and seasons (Fig. 4), indicating that leaf-miner communities in polyclonal plots were altogether more homogeneous than in monoclonal plots for which greater genotype-to-genotype variability was observed. At the within-plot level, the β-diversity (βsor) and the βsim component increased with an increasing genetic diversity (Table 3, Fig. 5). Similarly, the abundance variation (βbray) among trees within plot increased with an increasing genetic diversity (Table 3).

**Table 3:** Results of linear mixed-effect models testing for the effects of tree diversity, season, and year on within-plot β-diversity indices for both experiments

	Species diversity experiment				Genetic diversity experiment		
	*df*	*χ* ^2^	*P*		*df*	*χ* ^2^	*P*
*β*-*Diversity (βsor)*							
Species diversity	1	0.29	0.59	Genetic diversity	1	14.67	**< 0.001**
Season (S)	1	51.73	**< 0.001**	Season (S)	1	38.66	**< 0.001**
Year (Y)	1	0.65	0.42	Year (Y)	1	2.08	0.15
Area	2	1.06	0.59	S:Y	1	4.73	**0.029**
*Species turnover (βsim)*							
Species diversity	1	0.03	0.85	Genetic diversity	1	6.33	**0.012**
Season (S)	1	28.97	**< 0.001**	Season (S)	1	31.40	**< 0.001**
Year (Y)	1	37.88	**< 0.001**	Year (Y)	1	22.39	**< 0.001**
Area	2	0.23	0.89	S:Y	1	8.63	**0.003**
S:Y	1	5.20	**0.023**				
*Species nestedness (βnes)*							
Species diversity	1	0.07	0.79	Genetic diversity	1	0.07	0.80
Season (S)	1	0.43	0.51	Season (S)	1	3.67	0.055
Year (Y)	1	76.58	**< 0.001**	Year (Y)	1	32.66	**< 0.001**
Area	2	1.68	0.43	S:Y	1	4.71	**0.03**
S:Y	1	4.21	**0.04**				
*Abundance*-*based assemblage dissimilarity (βbray)*							
Species diversity	1	0.003	0.96	Genetic diversity	1	8.33	**0.004**
Season (S)	1	7.45	**0.006**	Season (S)	1	0.058	0.81
Year (Y)	1	14.96	**< 0.001**	Year (Y)	1	15.14	**< 0.001**
Area	2	1.18	0.55	S:Y	1	6.62	**0.010**
*Balanced variation in abundance (βbal)*							
Species diversity	1	0.13	0.71	Genetic diversity	1	3.30	0.069
Season (S)	1	15.43	**< 0.001**	Season (S)	1	7.40	**0.007**
Year (Y)	1	104.63	**< 0.001**	Year (Y)	1	62.52	**< 0.001**
Area	2	0.13	0.94				
*Abundance gradients (βgra)*							
Species diversity	1	0.33	0.56	Genetic diversity	1	0.005	0.94
Season (S)	1	8.25	**0.004**	Season (S)	1	18.82	**< 0.001**
Year (Y)	1	124.86	**< 0.001**	Year (Y)	1	67.85	**< 0.001**
Area	2	1.35	0.51				

Only significant interactions were kept in the final model. Significant effects (*P* < 0.05) are in bold

## Discussion

Our study compares the effects of tree species and genetic diversity on herbivores by considering plant diversity effects on both α- and β-diversity of herbivore communities within the same experimental context. We have demonstrated that both plant species and genetic diversity play important but different roles in structuring the associated herbivore communities at plot level. Tree species diversity affected abundance and species richness (α-diversity) of birch leaf miners, whereas tree genetic diversity influenced β-diversity of miner communities.

### Tree species diversity effects

Increase in the tree species diversity reduced birch leaf-miner abundance and miner richness in high tree density plots, while no effects of tree species diversity on species composition and β-diversity of leaf miners were found. This suggests that tree species richness has stronger effect on α-diversity of herbivores than on β-diversity. The observed reduction of leaf-miner abundance with plant species diversity contributes to the growing evidence across different ecosystems that the tree species diversity triggers a reduction of specialist insect herbivore abundance (Jactel and Brockerhoff 2007; Barbosa et al. 2009). The decrease in miner species richness might be the result of decreased miner abundance: as the number of mines decreases in mixed stands, the probabilities of these mines to include rarer miner species are also likely to decrease, resulting in a decreased miner species richness in diverse stands.

The associational resistance has been widely explained by a reduced resource concentration in mixtures, but the experimental studies have often failed to separate the effects of the diversity per see from the effects of the local density of the host plant (Underwood et al. 2014; Kim and Underwood 2015). Interestingly, in our experiment, we showed that associational resistance occurred only in plots with higher tree density, suggesting a density-dependent mechanism. While total tree density did not change the leaf-miner abundance, it altered the effects of tree species diversity on leaf-miner abundance in 2014. In contrast, we did not observe any significant tree species diversity effects on miner abundance in 2011 when all the plots were unthinned. The observed differences between the 2 years could have partly resulted from density-dependent patterns whereby tree diversity effects occurred only at high herbivore densities (Fernandez-Conradi et al. 2017), as leaf-miner abundance was four times higher in 2014 compared to 2011. Moreover, this difference was mainly due to higher abundance of specialist leaf miners, and only the abundance of specialist miners was affected by tree species diversity, while generalists were not. Hence, an increase in the proportion of specialist miners in 2014 might explain the difference in tree species diversity effects between the 2 years of survey. The observed temporal variation in the tree species diversity effect emphasizes the importance of long-term studies of plant diversity effects on herbivores (Barton et al. 2015).

### Birch genetic diversity effects

At the plot level, birch genetic diversity did not significantly affect miner abundance and species richness. This is likely to be due to the inconsistent responses across genotypes. Indeed, when birch genetic diversity effects were considered at genotype level, two birch genotypes have experienced associational resistance in mixed stands, i.e., a reduced miner abundance with increasing genetic diversity, whereas miner abundance on the other genotypes was unaffected. The two birch genotypes experiencing associational resistance were among the most susceptible to miners (i.e., had the highest miner abundance in monoclonal stands), suggesting that susceptible genotypes can get protection from miner attacks in mixed stands by being associated with more resistant neighbors. Yet, these genotype-dependent diversity effects occurred only in the early season. Similarly, Johnson et al. (2006) and Crutsinger et al. (2008) reported that genetic diversity effects on the associated herbivore community can change in magnitude and/or sign within a growing season. The seasonal shift in genetic diversity effect may have been mediated by leaf trait changes over the growing season. However, in a parallel study conducted in the species diversity experiment in 2014, the miner abundance on birch was not explained by any of the multiple leaf traits measured (Muiruri et al. 2018). Alternatively, the seasonal shift in the effect of genetic diversity might have been mediated by the changes in herbivore species composition. In our study, the species composition of birch leaf miners varied among seasons and years (data not shown) with communities dominated by different miner species at the different sampling dates. Because different miner species can respond differently to genetic diversity (Tack and Roslin 2011), variation in miner species composition may drive the observed variation in tree diversity effects.

The β-diversity analysis revealed that changes in miner species composition across plots or within plots were mainly driven by species turnover. Nestedness only played a minor role, suggesting that species-poor communities were not simply subsets of species-rich communities (Baselga 2010). Yet, the abundance dissimilarity across plots or within plots was mainly due to a balanced variation in abundance, whereas the gradient variation in abundance was negligible. In other words, lower miner abundance did not result from an overall reduced abundance of all the miner species, but was driven by the reduced abundance of some particular species. When these different components of β-diversity were split within mono and polyclonal plots, we found higher dissimilarity/turnover between monoclonal plots than between mixed stands. At the within-plot level, the turnover in miner species among trees increased with an increase in genetic diversity. Therefore, these findings suggest that each birch genotype supports a specific leaf-miner community and that mixing genotypes homogenized the composition of leaf-miner communities across plots.

## Conclusions

While the recognition of the importance of simultaneously considering plant species and genetic diversity effects on arthropods and ecosystem functioning is growing, most of the discussion so far focussed on whether plant genotypic diversity effects are of the same magnitude and direction as the effects of plant species diversity (Cook-Patton et al. 2011; Crawford and Rudgers 2013; Campos-Navarrete et al. 2015; Hahn et al. 2017). The results of our study demonstrate that plant species and genetic diversity effects on herbivores may affect different facets of herbivore diversity (α- vs β-diversity).

One caveat of our study is that we have independently manipulated tree species and birch genetic diversity and, hence, could not test for interaction between plant species and genetic diversity effects on herbivores. However, no interactive effects of plant genotypic and species diversity on herbivore abundance and species richness were found in studies which have manipulated genetic diversity of focal plant species both in the presence and absence of other plant species (Crawford and Rudgers 2013; Campos-Navarrete et al. 2015; Abdala-Roberts et al. 2015).

Our results have implications for conservation and management. Contrasting effects of plant species and genetic diversity on herbivores indicate that both aspects of plant diversity shape communities of associated herbivores, but in a different way. While maintaining tree species diversity might be important for maximizing total herbivore species richness per stand, α- and β-diversity of herbivores associated with a single tree species (i.e., birch) can be maximized by mixing different genotypes within a stand or by creating a mosaic of monocultures composed of different genotypes of the focal species. Moreover, we showed that genetic diversification of forest stands is unlikely to reduce the abundance of specialized herbivores at stand level, although it might help to protect more susceptible plant genotypes. On the other hand, species diversification of forest stands appears to be an effective way to reduce the densities of specialized herbivores, but only if the overall density of the stand is relatively high. Finally, our study showed the importance of long-term monitoring of the effects of plant diversity to take into account environmental factors that can lead to strong intra- and interannual variation. We propose that future studies focus not only on relative magnitude and direction of plant species and genetic diversity effects, but also integrate the interactions among tree and herbivore community characteristics such as diversity, density, and functional diversity.

## Electronic supplementary material

Below is the link to the electronic supplementary material.

Supplementary material 1 (PDF 143 kb)

## Data Availability

Data are available via  the Royal Holloway Figshare depository (DOI:10.17637/rh.7725158)
