# Supplementary material for: Contrasting effects of tree species and genetic diversity on the leaf-miner communities associated with silver birch
Source: Oecologia. 2019 Feb 24;189(3):687–97. doi: 10.1007/s00442-019-04351-x (PMC6418074; doi:10.1007/s00442-019-04351-x)
Supplement: Supplementary file 1 — Supplementary material 1 (PDF 143 kb) [file 442_2019_4351_MOESM1_ESM.pdf]

Online Resource 1. Species and genotype composition treatments in species diversity experiment (SDE) and genetic diversity experiment (GDE). In GDE, some treatments have been modified accordingly the results from genetic analyses (see material and methods). Only plots including birch in the SDE were selected.

Experiment	Species/genotype composition	Species/genotype richness
SDE	Birch	1
SDE	Alder	1
SDE	Larch	1
SDE	Pine	1
SDE	Spruce	1
SDE	Birch + Alder	2
SDE	Birch + Pine	2
SDE	Birch + Spruce	2
SDE	Pine + Larch	2
SDE	Pine + Spruce	2
SDE	Spruce + Larch	2
SDE	Spruce + Alder	2
SDE	Birch + Pine + Spruce	3
SDE	Birch + Pine + Larch	3
SDE	Birch + Pine + Alder	3
SDE	Birch + Larch + Alder	3
SDE	Pine + Spruce + Larch	3
SDE	Spruce + Larch + Alder	3
SDE	Birch + Pine + Spruce + Larch + Alder	5
GDE	O154	1
GDE	V5952	1
GDE	K5834	1
GDE	JR ¼	1
GDE	V5818	1
GDE	36	1
GDE	K2674	1
GDE	K1659 (+V5952)	1 + 1
GDE	36 + V5952	2
GDE	O154 + K5834	2
GDE	V5818 + V5952	2
GDE	36 + K1659 (+V5952)	2 + 1
GDE	V5818 + K1659 (+V5952)	2 + 1
GDE	JR ¼ + K2674 + O154 + K5834	4
GDE	JR ¼ + K2674 + V5952+36	4
GDE	O154 + K2674 + V5952 + V5818	4
GDE	JR ¼ + K5834+ K1659+36 (+V5952)	4+1
GDE	V5818+V5952+36+ K1659 (+V5952)	4+1
GDE	36+ K1659+ V5952+ O154+ K5834+ V5818+ JR ¼+ K2674	8

Online Resource 2. Genotypes of the eight birch genotypes used in the genetic diversity experiment at nine microsatellite loci.

Clone	L1.10	L2.2	L2.3	L2.7	L3.1	L3.4	L5.4	L13.1	L022
K5834	182/200	137/145	196/218	164/172	231/231	272/272	242/246	91/105	186/194
JR ¼	176/189	139/139	196/218	181/181	233/237	264/264	242/244	99/105	176/192
36	182/182	141/143	196/196	160/164	225/231	251/262	242/264	97/109	182/196
K2674	187/187	139/139	196/196	159/159	231/241	249/272	242/246	99/105	192/196
O154	182/189	139/141	196/196	164/191	231/233	262/265	242/242	99/103	178/194
V5818	176/207	137/143	196/218	175/191	231/233	261/272	242/244	99/99	192/194
K1659	182/191	139/141	196/196	160/160	231/231	264/264	242/254	97/99	178/200
V5952	187/187	137/137	196/214	160/181	229/231	261/261	242/246	117/117	180/184

Note: DNA from the three leaf samples of each genotype was extracted with the peqGOLD Plant DNA Mini Kit (PEQLAB, Erlangen, Germany). A multiplex PCR was performed using the QIAGEN Multiplex PCR kit with the following loci (fluorescent label of forward primers in brackets): L1.10 (Atto 550), L2.2 (Atto 550), L2.3 (FAM), L2.7 (Yakima Yellow), L3.1 (Yakima Yellow), L3.4 (FAM), L5.4 (Atto 550), L13.1 (FAM), L022 (Atto 565), L4.4 (Atto 565), L5.5 (Atto 565) and L11.1 (Yakima Yellow). Due to inconsistent amplification the last three loci could not be used for genotyping. Fragment analysis was performed on an ABI 3130xl genetic analyser resulting in a multilocus-genotype for each sample.

Online Resource 3. Species of leaf miners recorded in the Satakunta forest diversity experiments over the study period. Numbers indicate number of mines found in each of the experiments. GDE – genetic diversity experiment, SDE – species diversity experiment.

				**GDE**				**SDE**			
				**2011**		**2014**		**2011**		**2014**	
**Species name**	**Author**	**Order/Family**	**Diet breadth**	Early season	Late season	Early season	Late season	Early season	Late season	Early season	Late season
Agromyza alnibetulae	Hendel (1931)	Diptera, Agromyzidae	Polyphagous on deciduous trees	0	2	1	18	7	2	0	9
Anoplus plantaris	Naezén (1794)	Coleoptera, Curculionidae	Betula specialist	26	0	351	193	0	0	69	82
Anoplus roboris	Suffrian (1840)	Coleoptera, Curculionidae	Betula specialist	0	11	0	0	0	0	0	0
Bucculatrix demaryella	Duponchel (1840)	Lepidoptera, Bucculatricidae	Polyphagous on deciduous trees	36	255	12	411	8	212	7	134
Coleophora milvipennis	Zeller (1839)	Lepidoptera, Coleophoridae	Oligophagous on Betulaceae	0	0	13	5	0	0	1	2
Coleophora serratella	Linnaeus (1761)	Lepidoptera, Coleophoridae	Polyphagous on deciduous trees	404	105	441	68	177	55	136	74
Ectoedemia minimella	Zetterstedt (1839)	Lepidoptera, Nepticulidae	Betula specialist	5	26	2	1	3	9	6	5
Eriocrania spp.		Lepidoptera, Eriocraniidae	Betula specialist	216	0	784	0	84	0	516	0
Eriocrania sparmanella	Bosc (1791)	Lepidoptera, Eriocraniidae	Betula specialist	0	29	0	91	0	6	0	104
Fenusa pumila	Leach (1817)	Hymenoptera, Tenthredinidae	Oligophagous on Betulaceae	5	0	3	0	0	0	1	0
Heliozela hammoniella	Sorhagen (1885)	Lepidoptera, Heliozelidae	Betula specialist	0	0	0	2	0	0	0	1
Heterarthrus nemoratus	Fallén (1808)	Hymenoptera, Tenthredinidae	Betula specialist	0	15	0	74	0	5	0	4
Incurvaria pectinea	Haworth (1828)	Lepidoptera, Incurvariidae	Polyphagous on deciduous trees	271	9	50	9	180	7	49	5
Orchestes rusci	Herbst (1795)	Coleoptera, Curculionidae	Betula specialist	197	88	855	295	67	45	263	99
Orchestes testaceus	Müller (1766)	Coleoptera, Curculionidae	Betula specialist	2	0	1	0	0	0	14	1
Paronix betulae	Stainton (1854)	Lepidoptera, Gracillariidae	Betula specialist	1	4	101	194	4	9	18	100
Phylloporia bistrigella	Haworth (1828)	Lepidoptera, Incurvariidae	Betula specialist	0	11	0	100	0	5	0	9
Phyllonorycter cavella	Zeller (1846)	Lepidoptera, Gracillariidae	Betula specialist	0	1	0	10	0	17	0	7
Phyllonorycter ulmifoliella	Hübner, (1817)	Lepidoptera, Gracillariidae	Betula specialist	16	39	229	624	14	35	200	270
Profenusa thomsoni	Konow (1886)	Hymenoptera, Tenthredinidae	Betula specialist	0	28	0	48	0	19	0	8
Ramphus pulicarius	Herbst (1795)	Coleoptera, Curculionidae	Polyphagous on deciduous trees	0	36	0	82	0	30	0	0
Scolioneura betuleti	Klug (1816)	Hymenoptera, Tenthredinidae	Oligophagous on Betulaceae	0	55	0	66	0	37	0	34
Scolioneura vicina	Konow (1894)	Hymenoptera, Tenthredinidae	Betula specialist	0	0	0	0	3	0	0	0
Stigmella betulicola	Stainton (1856)	Lepidoptera, Nepticulidae	Betula specialist	0	3	0	417	0	8	0	25
Stigmella continuella	Stainton (1856)	Lepidoptera, Nepticulidae	Betula specialist	0	5	0	15	0	4	0	3
Stigmella lapponica	Wocke (1862)	Lepidoptera, Nepticulidae	Betula specialist	14	63	65	150	28	45	17	66
Stigmella luteella	Stainton (1857)	Lepidoptera, Nepticulidae	Betula specialist	0	18	0	47	0	0	0	3
Stigmella sakhalinella	Puplesis (1984)	Lepidoptera, Nepticulidae	Betula specialist	0	5	0	137	0	11	0	21
Unknown 1	n/a	n/a	*?*	7	0	0	0	11	14	0	0
Unknown 2	n/a	n/a	*?*	1	0	0	0	0	8	0	0
